# Lamprey Prohibitin2 Arrest G2/M Phase Transition of HeLa Cells through Down-regulating Expression and Phosphorylation Level of Cell Cycle Proteins

**DOI:** 10.1038/s41598-018-22212-0

**Published:** 2018-03-02

**Authors:** Ying Shi, Sicheng Guo, Ying Wang, Xin Liu, Qingwei Li, Tiesong Li

**Affiliations:** 1 College of Life Sciences, Lamprey Research Center, Liaoning Provincial Key Laboratory of Biotechnology and Drug discovery, Liaoning Normal University, Dalian, 116081 China; 2 210th Hospital of PLA, Dalian, 116011 China

## Abstract

Prohibitin 2(PHB2) is a member of the SFPH trans-membrane family proteins. It is a highly conserved and functionally diverse protein that plays an important role in preserving the structure and function of the mitochondria. In this study, the lamprey *PHB2* gene was expressed in HeLa cells to investigate its effect on cell proliferation. The effect of Lm-PHB2 on the proliferation of HeLa cells was determined by treating the cells with pure Lm-PHB2 protein followed by MTT assay. Using the synchronization method with APC-BrdU and PI double staining revealed rLm-PHB2 treatment induced the decrease of both S phase and G0/G1 phase and then increase of G2/M phase. Similarly, cells transfected with pEGFP-N1-Lm-PHB2 also exhibited remarkable reduction in proliferation. Western blot and quantitative real-time PCR(qRT-PCR) assays suggested that Lm-PHB2 caused cell cycle arrest in HeLa cells through inhibition of CDC25C and CCNB1 expression. According to our western blot analysis, Lm-PHB2 was also found to reduce the expression level of Wee1 and PLK1 and the phosphorylation level of CCNB1, CDC25C and CDK1 in HeLa cells. Lamprey prohibitin 2 could arrest G2/M phase transition of HeLa cells through down-regulating expression and phosphorylation level of cell cycle proteins.

## Introduction

Recently reports have suggested that cervical cancer (CC) represents one of the most common cancers among women worldwide^1,2^, accounting for over 500,000 new cases and 26000 cases of death annually^3,4^. Uncontrolled cell proliferation is an characteristic of tumor cells. Given that disruption of the cell cycle could have a major effect on cancer progression, a large number of studies have therefore tried to elucidate the molecular mechanisms of the cell cycle^5,6^. Thus cell cycle regulation and its modulation by various natural and synthetic agents have gained widespread attention in recent years.

Subsequently studies suggested various roles of PHBs in disease pathogenesis. Prohibitins comprises two subunits, PHB1 and PHB2, and both subunits are mainly localized in the mitochondrial inner membrane. They can assemble into a ring-like macromolecular structure, which plays a significant role in diverse intracellular processes, such as mitochondrial biogenesis, cell cycle progression and aging, as well as in many diseases, like obesity, diabetes and cancer^7^. PHBs can translocate into the nucleus or the mitochondria under apoptotic signals and the subcellular shuttling of prohibitin is necessary for apoptosis process^8^. PHBs are also involved in inflammatory diseases, such as inflammatory bowel diseases^9^. Therefore, PHBs are considered as important therapeutic targets for clinical applications^10^. In addition, PHB2 is an evolutionarily conserved protein that is ubiquitously expressed, and appears to be essential for cell survive in eukaryotes. PHB2 is mainly involved in the function of the mitochondrial inner membrane where it acts as a proteinlipid scaffold^11^. Some reports have also suggested that PHB2 plays a critical role in the regulation of E2F, pRb and p53^12^. In addition, PHB2 interacts with the cyclin-dependent kinase (CDK2), DNA repair associated enzymes and cell cycle associated proteins to influence multiple transcription factors and the cell cycle^13^. Its aberrant expression is closely related to cell carcinogenesis like breast, liver, ovarian, and thyriod cancers^14,15^. Lamprey is one of the most ancient vertebrates alive today, which makes it an excellent model for the study of vertebrate evolution, embryo development^16,17^, and the origin of adaptive immunity. It is also considered as a bridge that connects the invertebrates with the vertebrates. In contrast to the extensive studies of PHB2 from the mammalian, little work has been done on the PHB2 from *Lampetra morri* (*L*. *morri*). Until 2015, Li *et al*.^18^ firstly identified recombinant Lm-PHB2 (rLm-PHB2) protein could significantly enhance the H_2_O_2_-induced oxidative stress tolerance in Chang liver (CHL) cells. In this study, we found other functions of Lm-PHB2 that induces G2/M phase arrest in HeLa cells.

The eukaryotic cell cycle is tightly regulated and encompasses checkpoints in each of its different phases^19^. Cellular checkpoint control is pivotal in minimizing DNA damage accumulation and ensuring genomic integrity during cell cycle progression, that degregulation and resulting DNA damage have been implicated in many diseases, including cancer and neurodegenerative disorders^20^. Research conducted during the last two decades supports that nuclear cytoplasmic cycling of important G2 checkpoint proteins-such as cyclin-dependent kinase1 (CDK1), Cyclin B1, Wee1 kinase (Wee1), polo-like kinase 1 (PLK1) and cell division control protein 25C (CDC25C) is a key mechanism of G2 checkpoint regulation^21,22^.

In this study, Lm-PHB2 was found to inhibit the proliferation of HeLa cells through down-regulating the expression level of CCNB1, CDC25C, Wee1 and PLK1. Similarly, phosphorylation level of CCNB1, CDC25C and CDK1 has been inhibited, then leading to G2/M phase arrest and cell apoptosis, suggesting that Lm-PHB2 may have therapeutic value in the treatment of cervical cancer.

## Results

### Lm-PHB2 inhibits HeLa cells proliferation

**Figure 1:**
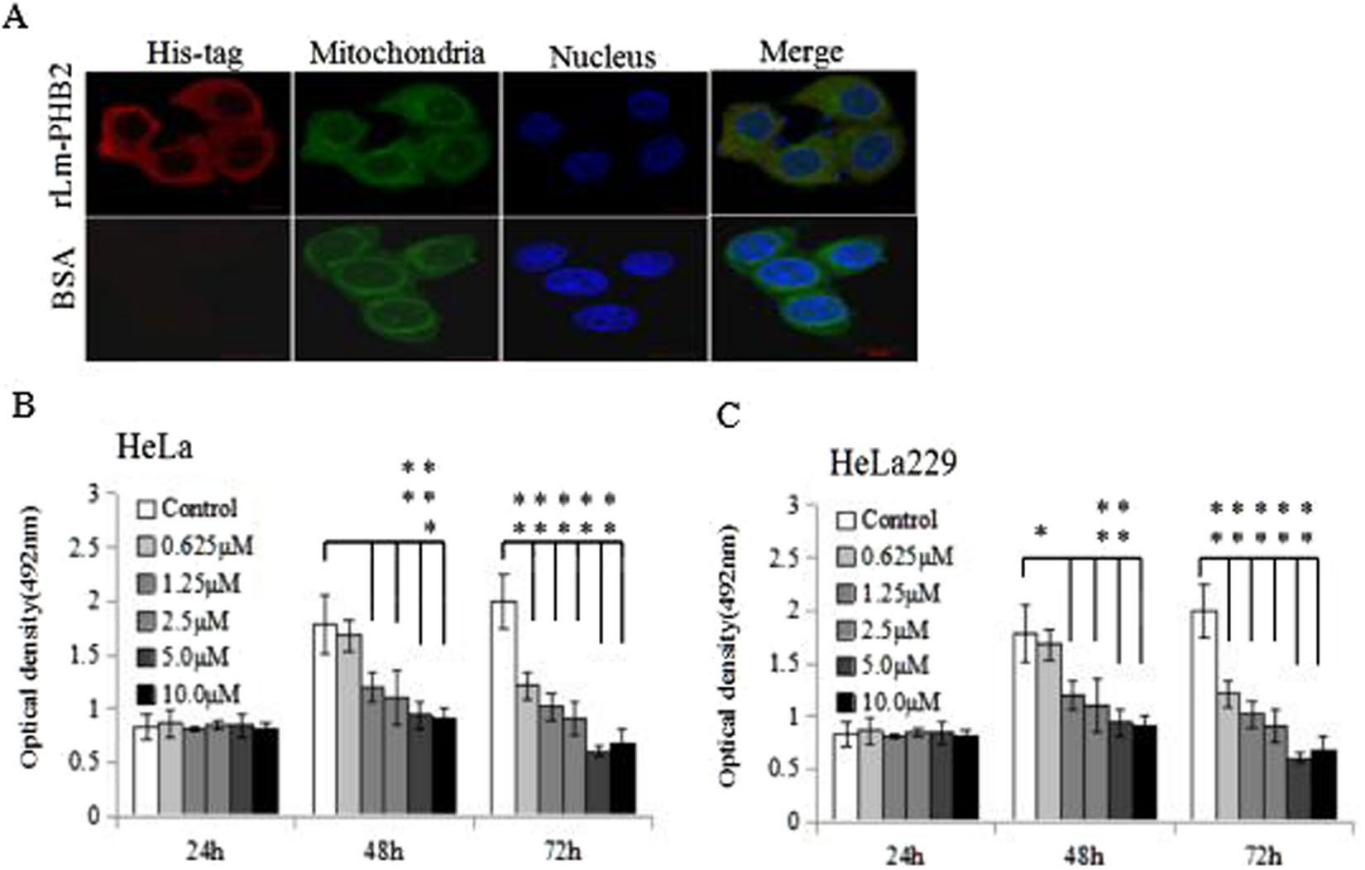
Effect of purified rLm-PHB2 on the proliferation of human cervical cancer cells. (**A**) Confocal microscopic images of rLm-PHB2 protein entering into HeLa cells and localizing in cytoplasm. rLm-PHB2 culture 24 h were immunostained with His-tag antibody (Red). Mitochondria were stained with MitoTracker (Green), and nucleus were stained with Hoechst33258 (bule). Scale bar, 10 μm. (**B**,**C**) HeLa and HeLa 229 cells were treated with PBS or different concentrations of purified Lm-PHB2 (0.625 μM, 1.25 μM, 2.5 μM, 5.0 μM and 10.0 μM) at 37 °C for 24 h, 48 h or 72 h and cell viability was determined by the MTT assay. Data are the means ± SDs from three experiments, each carried out in triplicate. ‘*’ and ‘**’ indicate significantly different from control cells (not treated with Lm-PHB2) at the *P* < 0.05 and *P* < 0.01 levels, respectively.

To investigate the anti-proliferative effect of Lm-PHB2, two HeLa cell lines were treated with various concentrations of rLm-PHB2 for different periods of time followed by cell viability assay. Exogenous rLm-PHB2 protein with His-tag was mostly localized in cytoplasm in HeLa and C33A cells (Figs 1A, S1). In both HeLa and HeLa299 cells lines, the untreated cells (PBS-treatment only) increased their number at a faster rate than their rLm-PHB2-treated counterparts, and this was evident in all three time points measured (Fig. 1 B,C). In both cases, reduction in cell growth caused by PHB2 was significant for all concentrations of PHB2 tested after 48 h of treatment, except for those treated with 0.625 μM rLm-PHB2 proteins. However, in the case of 72 h treatment, all concentrations of PHB2 caused significant reduction.

**Figure 2:**
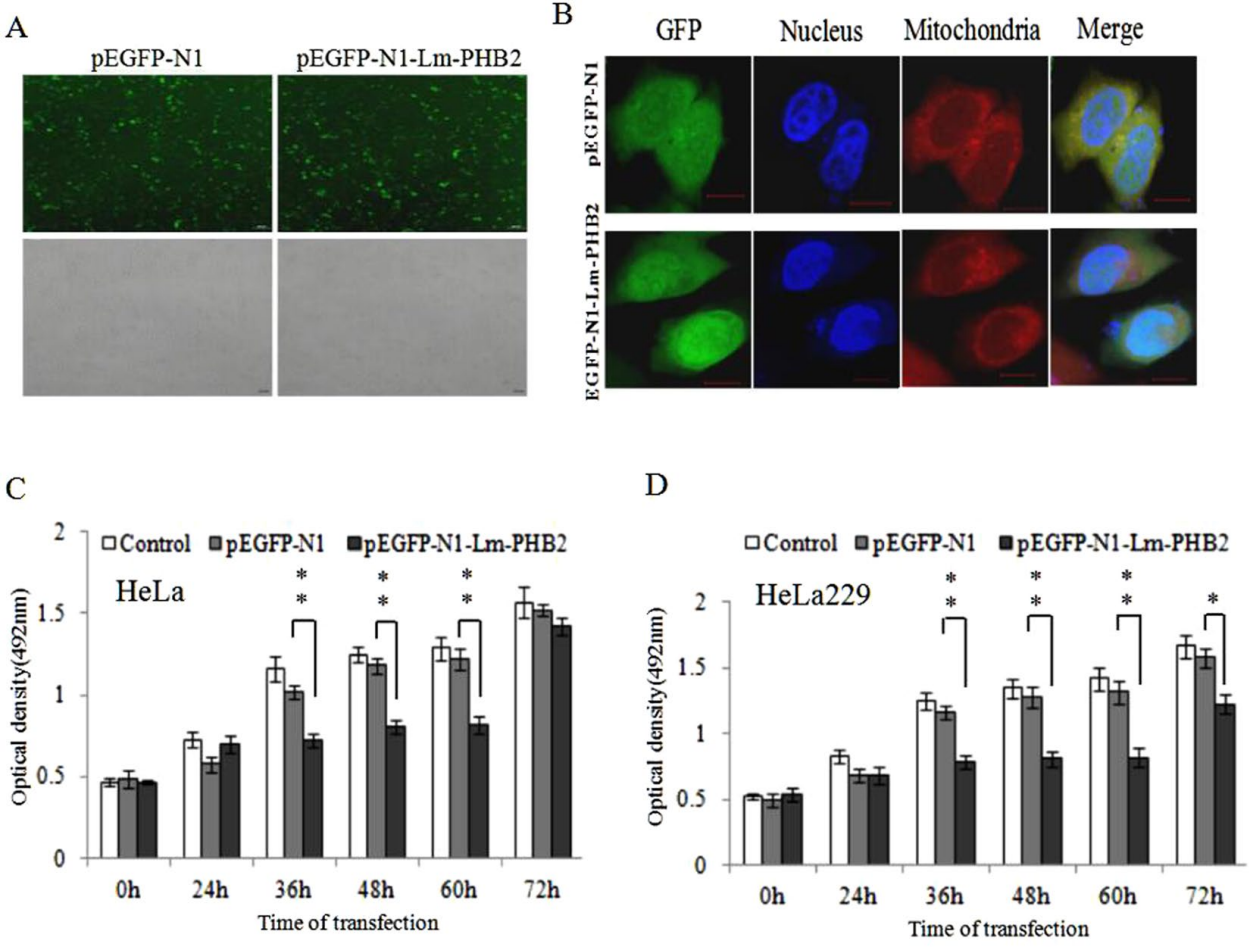
Effect of overexpression of Lm-PHB2 on the proliferation of human cervical cancer cells. (**A**)Transfer efficiency of HeLa cells after transfected with pEGFP-N1-Lm-PHB2 or pEGFP-N1 at 37 °C for 24 h. Scale bar, 100 μm. (**B**) Subcellular organelles localization of Lm-PHB2 protein in HeLa cells visualized by confocal microscopy with Hoechst33258 (blue, nucleus), GFP (green, Lm-PHB2), and MitoTracker Red (red, mitochondria). Scale bar, 10 μm. (**C**,**D**) HeLa and HeLa 229 cells were transfected with pEGFP-N1-Lm-PHB2 or pEGFP-N1 at 37 °C for 24 h, 36 h, 48 h, 60 h or 72 h and the viability of the cells was determined by MTT assay. Data are the means ± SDs from three experiments, each carried out in triplicate.‘*’and ‘**’ indicate significantly different from cells transfected with pEGFP-N1 at the *P* < 0.05 and *P* < 0.01 levels, respectively.

Similar result was obtained when these two cells lines were transfected with a plasmid containing Lm-PHB2 (pEGFP-N1-Lm-PHB2) instead of being treated with the protein (Fig. 2A). In addition, GFP-fused Lm-PHB2 could be expressed and was mostly localized in nucleus, a small amount in the cytoplasm and mitochondria in HeLa and C33A cells (Figs 2B, S2). Cells that were transfected with pEGFP-N1-Lm-PHB2 showed significantly reduced growth compared to cells transfected with pEGFP-N1 for HeLa cells. Growth reduction occurred most prominently from 36 to 60 h after transfection in the case of HeLa cells, or 36 to 72 h post transfection in the case of HeLa 299 cells (Fig. 2C,D). Thus expression of Lm-PHB2 in these cells had the same growth inhibitory effect as treatment of these cells with the rLm-PHB2 protein.

### Lm-PHB2 induced G2/M phase cell cycle arrest

**Figure 3:**
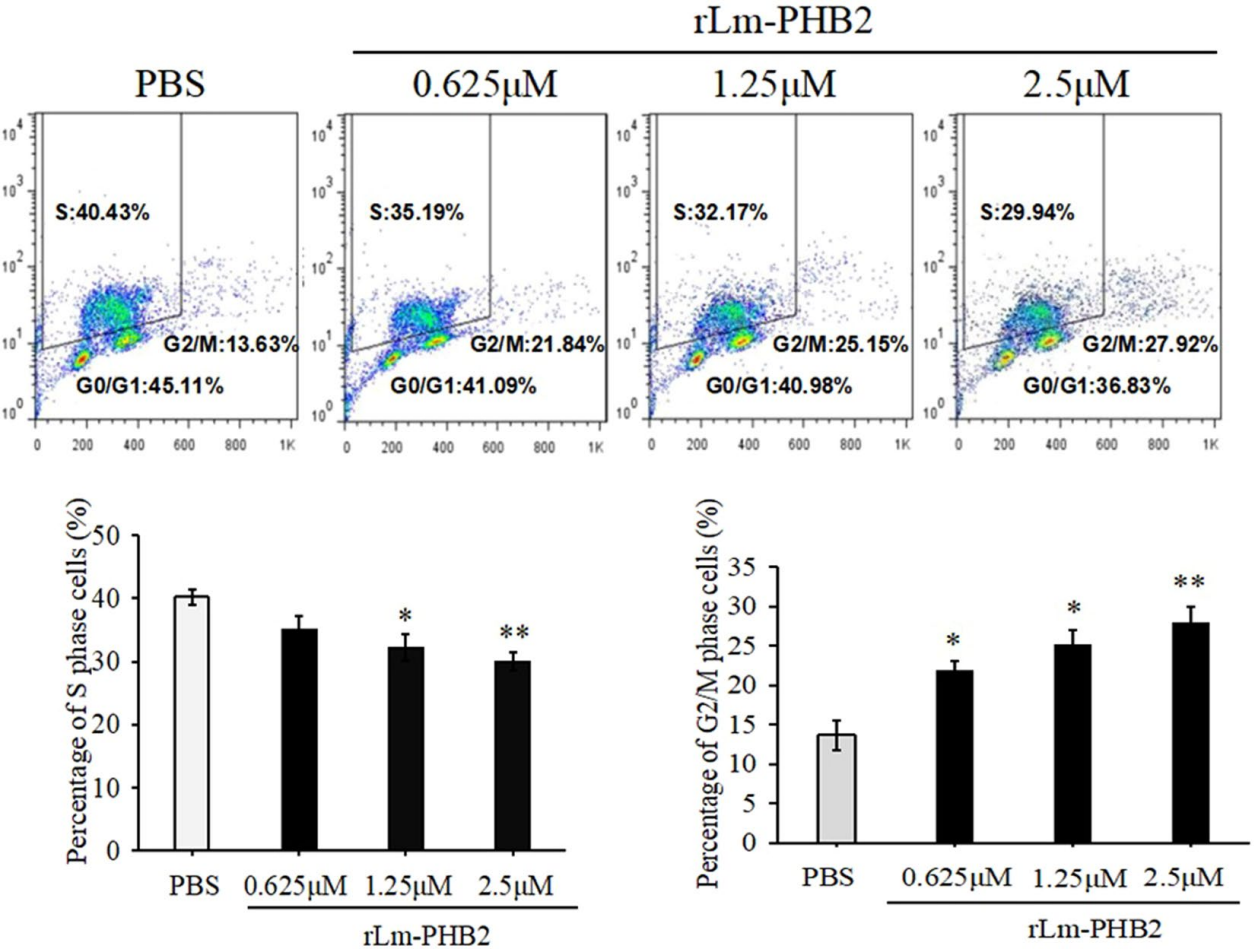
Effect of Lm-PHB2 on the cell cycle. The double staining with both APC-BrdU and PI for detection of G1/S and/or G2/M cell cycle alteration after HeLa cells treated with rLm-PHB2 protein and PBS treatment as control.

**Figure 4:**
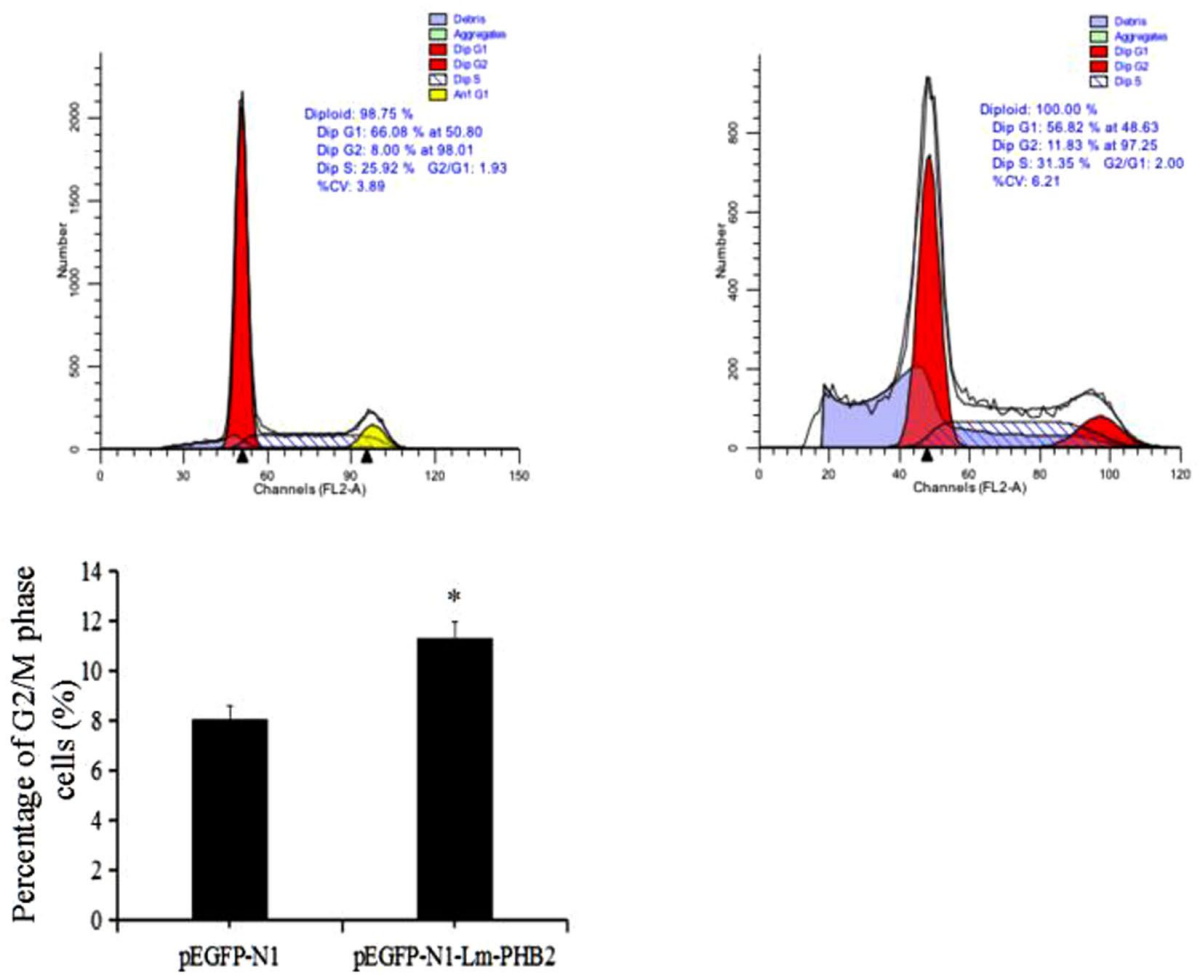
Effects of Lm-PHB2 on the proportion of cells in different phases of the cell cycle. HeLa cells were transfected with pEGFP-N1 or pEGFP-N1-Lm-PHB2 at 37 °C for 36 h followed by PI staining and flow cytometry analysis. The histogram shows the percentage of G2/M phase cells in both groups. Data are the means ± SDs from three experiments. ‘*’ indicates significantly different from PBS-treated cells at the *P* < 0.05 level.

In order to better understand the mechanism of growth inhibition exerted by Lm-PHB2 on HeLa cells, the percentage of cells cycle in the presence of rLm-PHB2 protein was analyzed using double staining with both APC-BrdU and PI. The results showed that rLm-PHB2 treatment induced the decrease of both S phase and G0/G1 phase and then increase of G2/M phase (Fig. 3). In addition, the results of PI staining only showed that compared to the PBS-treated group, the percentage of G2/M phase cells in Lm-PHB2-treated groups was increased, and in an Lm-PHB2 concentration-dependent manner (Supplementary 3). Similarly, HeLa cells transfected with pEGFP-N1-Lm-PHB2 also exhibited significant increases in the proportion of G2/M phase cells compared to those transfected with pEGFP-N1 (Fig. 4). Thus, Lm-PHB2 appeared to inhibit the growth of HeLa cells by inducing cell cycle arrest at the G2/M phase. Since it was easier to observe the experimental phenomena in HeLa cells than in HeLa 229 cells, subsequent experiments therefore focused on HeLa cells only.

### Lm-PHB2 induces cell cycle arrest through down-regulating the expression and phosphorylation level of cell cycle proteins

**Figure 5:**
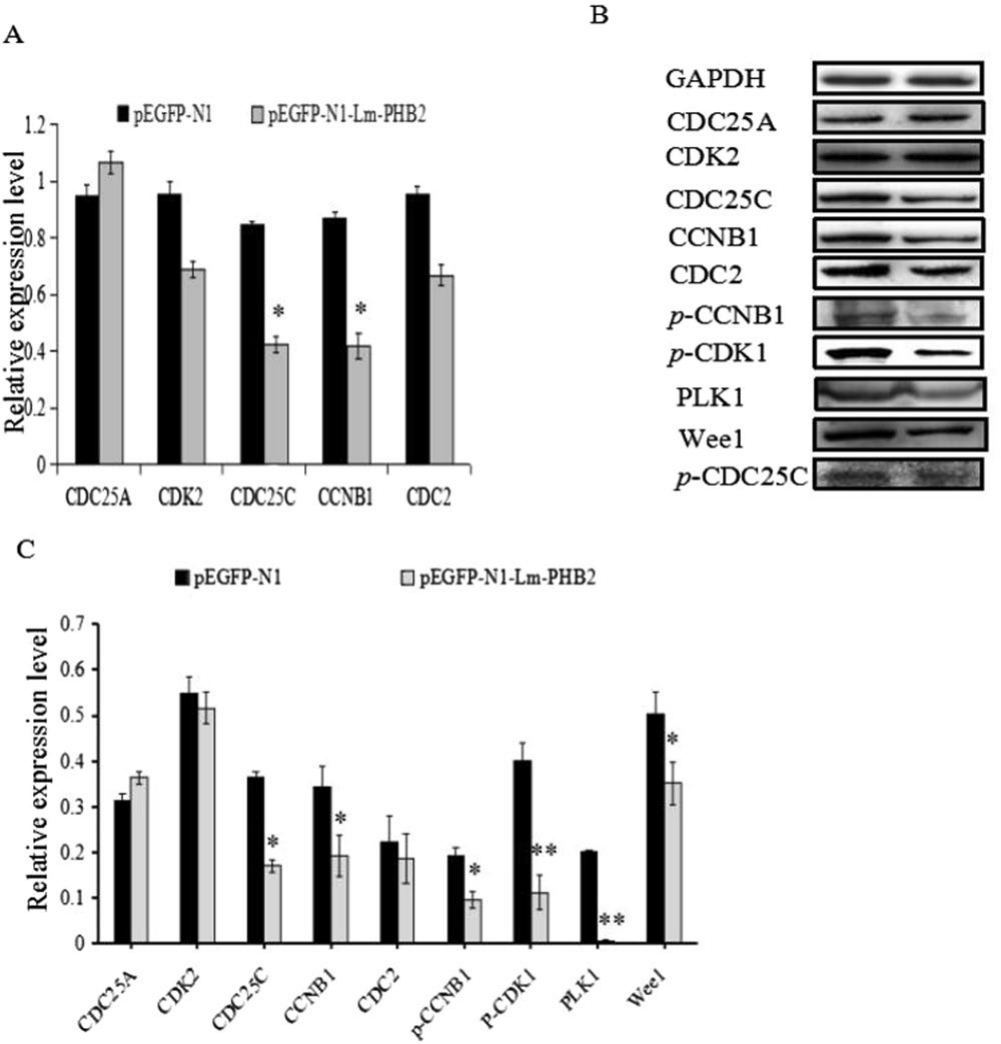
Lm-PHB2 induces cell cycle arrest through down-regulating the expression and phosphorylation level of cell cycle proteins. (**A**,**B**) Changes in relative expression levels of cell cycle proteins determined by *q*RT-PCR and western blot. (**C**) The histogram in B compares the intensities of the various bands shown in the blot. Data in the histograms are the means ± SDs from three determinations. ‘*’ and ‘**’ respectively indicates significantly different from PBS-treated cells at the *P* < 0.05 or *P* < 0.01 level.

To examine the possible mechanism associated with G2/M phase arrest induced by Lm-PHB2, the effect of Lm-PHB2 on the expression of G2/M phase proteins expression and phosphorylation level was investigated. HeLa cells were transfected with pEGFP-N1-Lm-PHB2 or pEGFP-N1 for 48 h, and the transcript and protein levels of these genes were quantified by real-time PCR and western blot, respectively. Cells transfected with pEGFP-N1-Lm-PHB2 showed significantly reduced expression of CDC25C and CCNB1, both at the transcript (Fig. 5A) and protein (Fig. 5B) levels compared to cells transfected with pEGFP-N1. As for CDC25A, CDK2 and CDC2, no change in either transcript or protein level was found between cells transfected with pEGFP-N1-Lm-PHB2 and pEGFP-N1. Subsequently, according to western blot analysis, Lm-PHB2 was also found to reduce the expression level of Wee1, PLK1, *p*-CDC25C, *p*-CCNB1 and *p*-CDK1 in HeLa cells. These results strongly suggested that Lm-PHB2 induced G2/M phase cell cycle arrest through down-regulating the expression and phosphorylation level of cell cycle proteins.

## Discussion

### Lamprey PHB2 induces HeLa cells apoptosis

Prohibitin 1 (PHB1) and prohibitin 2 (PHB2) are the two highly homologous subunits of the eukaryotic mitochondrial PHB complex^21^. A large number of studies have shown that prohibitin can translocate into the nucleus or the mitochondria under apoptotic signals and the subcellular shuttling of prohibitin is necessary for apoptosis process. In the nucleus, PHB regulates transcriptional activation and cell cycle. At the mitochondrial inner membrane that implicated in mitochondrial genome stabilization, mitochondrial morphology, oxidative stress, and apoptosis^16,22^. Moreover, increased membrane localization of PHBs and the PHB1/c-Raf complex in activated hepatic stellate cells may promote Tan IIA-induced apoptosis^8,23^. To our knowledge, there has been no report concerning lamprey PHB2 in cervical cancer. Recently, lampreys are considered to be the most scientifically accessible model of the remaining jawless vertebrates. In the present study, lamprey PHB2 was found to inhibit the proliferation of HeLa cells through arresting cell cycle transition. The results we obtained before showed the cell cycle arrest induced by Lm-PHB2 was not creative by protein antigenicity against human. We also found the number of S phase cells decreased after treatment with rLm-PHB2 (Supplementary 3). It is difficult that this altered G1/S phase state is caused by which rLm-PHB2 functioned against G1/S and/or G2/M transition. So the alteration of G1/S and/or G2/M transition was examined using double staining with both APC-BrdU and PI after HeLa cells treated with rLm-PHB2. This result revealed that rLm-PHB2 treatment induced the decrease of both S phase and G0/G1 phase and then increase of G2/M phase (Fig. 3).

### Lamprey PHB2 arresting the cell cycle at the G2/M phase

Cell-cycle checkpoints at the G2/M as well as G1/S phases are critical for maintaining DNA integrity and regulating the passage of cells through the cell cycle^24^. It is well known that the loss of these checkpoints can lead to the transition and progression of cancer cells. CDC25C is responsible for stimulating and maintaining the complexes CCNB1-CDK1 activation that ultimately determines to pass the G2 checkpoint^25^. PLK1 also promotes G2/M transition progression through affecting the subcellular localization of CDK1 regulatory checkpoint nodes. Cytoplasmic activity of PKL1 is also required to prime the CCNB1-CDK1 complex for nuclear localization by phosphorylation of CCNB1. Moreover, Wee1 also regulate G2/M phase transition and cell mitosis through phosphorylation of CDK1 and down-regulation of CDC2 kinase activity^26^. This means that when the expression level of CCNB1, CDC25C, CDK1 and PLK1 is reduced, the abilities in tumor cells proliferation could be inhibited at the same time. According to expression level analysis of cell cycle proteins, lamprey PHB2 was found to reduce the expression level of CCNB1, CDC25C, Wee1, CDK1 and PLK1 in HeLa cells, which indicated that lamprey PHB2 could arrest the G2/M phase transition processes of HeLa cells via affecting the down-regulation expression level of CCNB1, CDC25C, Wee1, CDK1 and PLK1. Similarly, phosphorylation level of CCNB1, CDC25C and CDK1 has been down-regulated in HeLa cells. Activated PLK1 could phosphorylate CDC25C on Ser216 and CCNB1 on serine residues and promote cytoplasmic-nuclear translocation of CDC25C^27^. In the nucleus, Wee1 could phosphorylate CDK1 on Tyr15 and phosphorylated CDK1 inactivates the kinase and thus induces G2/M phase transition arrest^28,29^. Above results indicated that lamprey PHB2 could induce cell cycle arrest not only through inhibiting expression level of CCNB1, CDC25C, Wee1, CDK1 and PLK1, but also through down-regulation phosphorylation level of CCNB1, CDC25C and CDK1 in HeLa cells. In order to meet the criterion of genetic engineering drugs, overcoming antigenicity against human and removing the His-tag from rLm-PHB2 would provide the opportunity for the application of rLm-PHB2 as a potential anti-tumor drug in the future clinical studies.

## Materials and Methods

### Materials

DMEM, trypsin-EDTA solution, paraformaldehyde, Triton-X 100, Hoechst33258 and 2,5-diphenyltetrazolium bromide (MTT) were purchased from Beyotime Biotechnology (Beijing, China). Cell cycle detection kit and BrdU cell proliferation detection kit was purchased from KeyGEN BioTECH. Fetal bovine serum was purchased from Gbico (USA). Lipfectamine 2000 Reagent was obtained from Invitrogen (Thermo Fisher Scientific, US); Reverse Transcription Kit and SYBR Premix ExTaq^TM^II Kit were purchased from TaKaRa (Dalian, China). SanPrep EndoFree Plasmid Kits, antibody of CDK1, CDK2, CDC25A, CDC25C, CCNB1, PLK1, Wee1, *p*-CDK1 (phospho Tyr15) and GAPDH and PCR primers were obtained from Shangon Biotech (Shanghai, China). Monoclonal mouse antibody *p*-CCNB1 (phospho Ser147), *p*-CDC25C (phospho Ser216), anti-rabbit IgG-conjugated Alexa 594 antibody and anti-His-tag antibody were obtained from Proteintech Company (Wuhan, China).

### Cloning, expression, and purification of the lamprey PHB2 protein

According to our previous study, a termination codon was added to the end of the open reading frame (ORF) of Lm-PHB2. The Lm-PHB2 gene was amplified from *Lamprey morii* (Chinese northeast lamprey) cDNA library (prepared from the cardiac muscle) with forward primer (5′-GGAATTCCATGGCTCAGCTCAAGGA-3′; underlined bases indicate *Eco*RI site) and reversed primer (5′-CCCAAGCTTGGGCTTCTTTTTCACCGAC-3′; underlined bases indicate *Hind*III site). The amplified DNA fragment was subsequently digested with *EcoRI* and *Hind*III and then cloned into *EcoR*I-*Hind*III cut pET32a to yield the construct pET32a-Lm-PBH2, which was introduced into *E*. *coli* BL21 (DE3) where Lm-PHB2 was expressed as a His-tagged protein and purified by Ni-NTA affinity chromatography. The soluble fraction of the cell extract was applied to a 1-ml Ni-NTA column pre-equilibrated with binding buffer (20 mM Tris-HCl (pH 8.0)/ 500 mM NaCl/20 mM imidazole). After washing the column with wash buffer (20 mM Tris-HCl (pH 8.0)/500 mM NaCl/30 mM imidazole), the bound Lm-PHB2 was eluted with elution buffer (20 mM Tris-HCl (pH 8.0)/500 mM NaCl/80 mM imidazole). The concentration of Lm-PHB2 was measured using a bicinchoninic acid (BCA) protein assay kit. The purified Lm-PHB2 was analyzed by SDS-PAGE and stored at −80 °C.

### Cell culture

HeLa cell lines were from stocks preserved in our laboratory. The cells were grown in DMEM medium supplemented with 10 % fetal bovine serum and in a 37 °C humidified incubator with 5 % CO_2_. The cells were grown to 70 % confluence and then harvested by digestion with trypsin-EDTA, and further plated in 6-well (2 × 10^5^ cells/well) or 96-well plates (1 × 10^4^ cells/well) for subsequent experiments.

### Immunofluorescence

The HeLa cells (1 × 10^5^) were cultured on slides in 24-well plates for 24 h, and then treated with 10 μM rLm-PHB2 or 10 μM Bovine Serum Albumin (BSA) for 24 h. After rinsing with PBS, the HeLa cells were fixed with 4 % paraformaldehyde for 15 min. 1 % Triton X-100 treated with Hela cells for 15 min, and then blocked with BSA and incubated with His-tag antibodies (1:1000) for 3 h at room temperature. Secondary antibodies (1:5000) incubated for 40 min. Subsequently, the HeLa cells were washed with PBS twice, and then stained with Mitotracker (Green) 3 min at room temperature in the dark. After rinsing with PBS twice, stained with Hoechst 33258 for 3 min at room temperature in the dark. After washing with PBS twice, a laser scanning confocal microscopy was used to observe the HeLa cells at 630× magnification.

### Cell proliferation assay

HeLa cells were grown in 96-well plates (1 × 10^4^ cells/well) in DMEM supplemented with 10 % FBS. After removing the medium, the cells were treated with phosphate buffered saline (PBS) or different concentrations of Lm-PHB2 (from 0.625 μM to10.0 μM) for 24 h. Cell viability was then determined by MTT assay and expressed as percentage relative to control cells (HeLa cells treated with PBS only).

### Flow cytometry analysis

HeLa cells were grown in 6-well plates (2 × 10^5^ cells/well) for 12 h and then straved without serum for 16 h, treated with PBS or rLm-PHB2 at 0.625 μM, 1.25 μM and 2.5 μM for 24 h. BD Biosciences APC BrdU Flow kit (cat # KGA319-03) was used for analysis. BrdU (10 uM) was added to culture medium and cells were incubated for an additional 4 hours, then harvested with 0.25 % trypsin and 0.1 % EDTA in HeLa cells and fixed and stained according to the protocol provided by the BD Biosciences APC BrdU Flow kit and analysized on flow cytometer. Analysis was performed using Flow jo 7.6.1 software.

HeLa cells were grown in 6-well plates (2 × 10^5 ^cells/well) for 12 h, and then transfected with pEGFP-N1 or pEGFP-N1-Lm-PHB2 plasmid for 24 h. After removing the medium, the adherent cells were digested with ethylene diaminetetraacetic acid (EDTA) free trypsin (HyClone, USA) and harvested by centrifugation. The cells were then washed twice with PBS and fixed in 500 μL ice-cold 70 % ethanol at 4 °C for 2 h. The fixed cells were then centrifuged at 2000 × g for 2 min and the pellet was washed with PBS. After that, the cells were resuspended in 100 μL RNase I and incubated at 37 °C for 30 min followed by the addition of 400 μL propidium iodide and further incubation at 4 °C in the dark. The sample was finally analyzed by flow cytometry.

### Cell transfection

Transfection using Translipid Transfection Reagent (TransGen Biotech, China) was performed according to the manufacturer’s instructions on cells that were at least 70 % confluent. After 24 h, the cells were washed with PBS, and the medium was replaced with fresh, normal growth medium with or without 250 mM H_2_O_2_ for 3 h for subsequent MTT assays, *q*RT-PCR and western blotting.

### Quantitative real-time PCR (*q*RT-PCR)

HeLa cells were transfected with the pEGFP-N1 or pEGFP-N1-Lm-PHB2 for 36 h and total RNA was then isolated from the cells using RNAiso Plus (TAKARA, China). The RNA was subjected to reverse transcription using the PrimeScript^TM^ RT reagent Kit with gDNA Eraser (TAKARA, China). Quantitative RT-PCR was performed with the SYBR Premix ExTaq^TM^II Kit (TAKARA, China) according to the manufacturer’s protocol using GAPDH as an internal control. Quantitative real-time PCR used primers used are listed in Table 1.

**Table 1:** Primers used for Quantitative Real-time PCR.

Primers name	Primers sequence
GAPDH forward	5′-CAGGAGGCATTGCTGATGAT-3′
GAPDH reverse	5′-GAAGGCTGGGGCTCATTT-3′
CDC25A forward	5′-GCACTCGGTCAGTGTTGAAG-3′
CDC25A reverse	5′-CATGGGCCTTCTCTGGATTA-3′
CDC2 forward	5′-AGTGTGGCCAGAAGTGGAAT-3′
CDC2 reverse	5′-TTTCGAGAGCAAATCCAAGC-3′
CDK2 forward	5′-GTGGTGTGGCCAGGAGTTAC-3′
CDK2 reverse	5′-CGATAACAAGCTCCGTCCAT-3′
CCNB1 forward	5′-CAAGCCCAATGGAAACATCT-3′
CCNB1 reverse	5′-GGATCAGCTCCATCTTCTGC-3′
CDC25C forward	5′-TGTCAACCCAGAAACAGTGG-3′
CDC25C reverse	5′-CTGGATGTGTCCTCCCAGAT-3′

### Western blot

HeLa cells were seeded into 6-well plates (1 × 10^5^ cells/well) and incubated at 37 °C for 24 h. The cells were subsequently transfacted with pEGFP-N1 or pEGFP-N1-Lm-PHB2 plasmids for 24 h at 37 °C. After that, the cells were harvested by centrifugation and lysed in cell lysis buffer containing 0.2 mM pheylmethanesulfonyl fluoride (PMSF). The cell lysate was then centrifuged at 5000 × g for 15 min and the supernatant was retained. Total protein concentration in the supernatant was measured by the BCA Kit using BSA as a standard. The samples was then subjected to SDS-PAGE using 12 % gel. After electrophoresis, the proteins in the gel were transferred to a polyvinylidene difuoride (PVDF) membrane. The membrane was blocked with 5 % non-fat dairy milk in TBST buffer (20 mM Tris-HCl (pH 8.0)/150 mM NaCl/0.05 % Tween-20) for 2 h, and then incubated with the appropriate primary antibody at room temperature for 5 h. Antibody of CDC25A, CDK2, CDC25C, CCNB1, CDC2, p-CDK1, PLK1, Wee1 and GAPDH was used at 1:500 dilution, whereas antibody of *p*-CCNB1 and *p*-CDC25C was used at 1:1000 dilution. After incubation with the primary antibody, the blot was washed five times with TBST buffer followed by incubation with horseradish peroxidase-conjugated goat anti-rabbit secondary antibody at 1:5000 dilution for 1 h at room temperature. The blot was again washed five times with TBST buffer and finally visualized with the BeyoECL Plus detection Kit. Specific protein bands detected via immunoblot analysis were quantified via densitometry (Gel-Pro analyzed 4). The scanned image was inverted to measure the gray value of a specific protein band. The numerical value was recorded as an gray value. The histograms were shown to indicate the changes of the relative cell cycle proteins level in HeLa cells after transfacted with pEGPF-N1 or pEGFP-N1-Lm-PHB2 plasmids control with GAPDH.

### Statistical analysis

Statistics’ t-test was used to analyze differences between test groups and control groups. Statistical significance was considered at the *P* < 0.05 and or *P* < 0.01 level.

### Data availability

All data generated or analysed during this study are included in this published article (and its Supplementary Information files). Raw datasets generated are available from the corresponding author on reasonable request.

## Electronic supplementary material

Supplementary Information
