# Supplementary material for: Lamprey Prohibitin2 Arrest G2/M Phase Transition of HeLa Cells through Down-regulating Expression and Phosphorylation Level of Cell Cycle Proteins
Source: Sci Rep. 2018 Mar 2;8:3932. doi: 10.1038/s41598-018-22212-0 (PMC5834496; doi:10.1038/s41598-018-22212-0)
Supplement: Supplementary file 1 — Supplementary Information [file 41598_2018_22212_MOESM1_ESM.doc]

**Lamprey Prohibitin2 Arrest G2/M Phase Transition of HeLa Cells through Down-regulating Expression and** **Phosphorylation Level of Cell Cycle Proteins**

*Ying Shi^1^, Sicheng Guo^1^,Ying Wang ^2^, Xin Liu^1^, Qingwei Li 1[[1]](#footnote-2)*, Tiesong Li1[[2]](#footnote-3)**

*(1.College of Life Sciences,Lamprey Research Center,Liaoning Provincial Key Laboratory of Biotechnology and Drug discovery, Liaoning Normal University, Dalian 116081, China; 2.**210th HospitaI of PLA, DaIian 116011, China)*

Exogenous rLm-PHB2 protein with His-tag was mostly localized in cytoplasm in C33A cells. Confocal microscope analysis show that rLm-PHB2 proteins mainly located in cytoplasm and a small amount located in the nucleus.


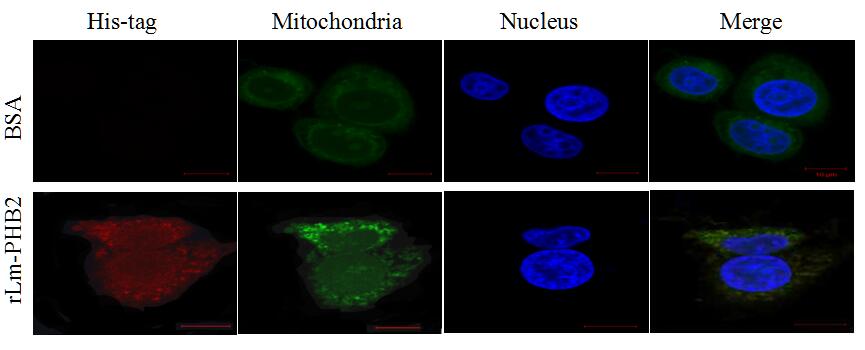


**Supplementary 1.** Confocal microscopic images of rLm-PHB2 protein entering into C33A cells and localizing in cytoplasm. rLm-PHB2 culture 24 h were immunostained with His-tag antibody(Red). Mitochondria were stained with MitoTracker(Green), and nucleus were stained with Hoechst33258(bule). Scale bar, 10 μm.

In addition, GFP-fused Lm-PHB2 could be expressed and was mostly localized in the cytoplasm and mitochondria , a small amount in nucleus in C33A cells.

**
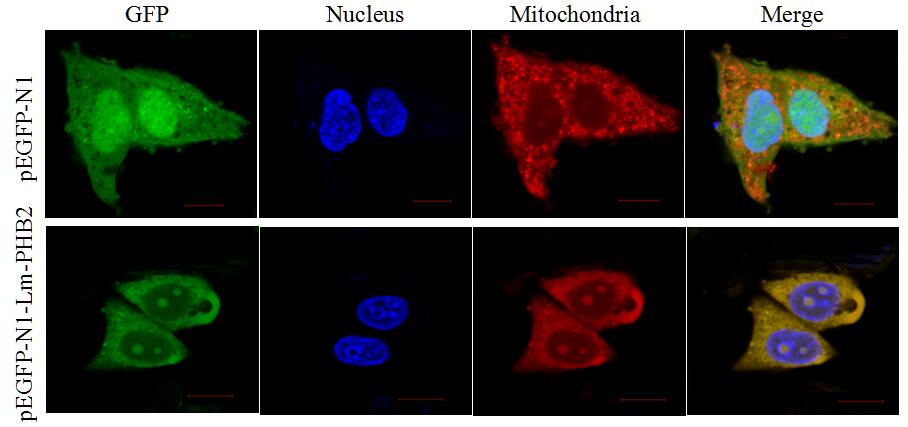
**

**Supplementary 2.** Subcellular organelles localization of Lm-PHB2 protein in C33A cells visualized by confocal microscopy with Hoechst33258 (blue, nucleus), GFP (green, Lm-PHB2), and MitoTracker Red (red, mitochondria). Scale bar, 10 μm.

In order to prove alterations of G2/M or G1/S transition induced by rLm-PHB2 proteins but not by other factors. HeLa cells were starved without serum for 16 h, 18 h or 20 h, and then using PI staining assay. Flow cytometry analysis results showed synchronized cell cycle was kept at G0/G1 phase, no significant difference in the treatment time course.


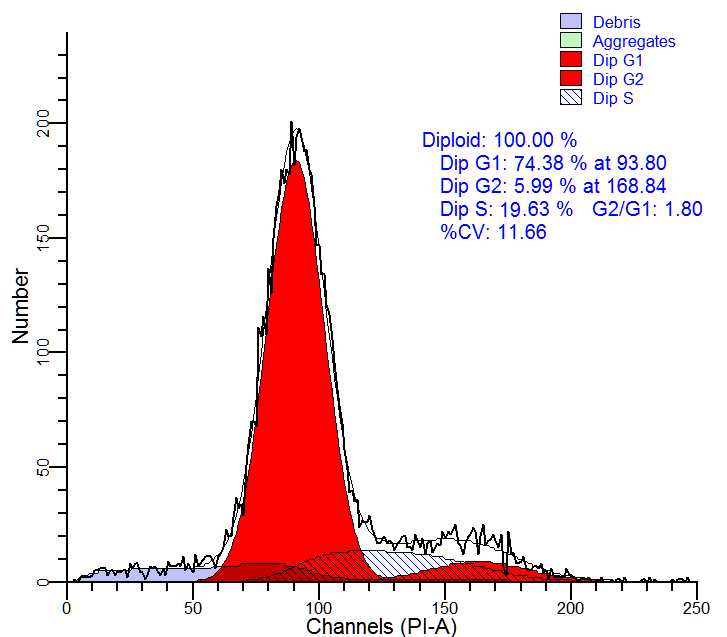


18 h


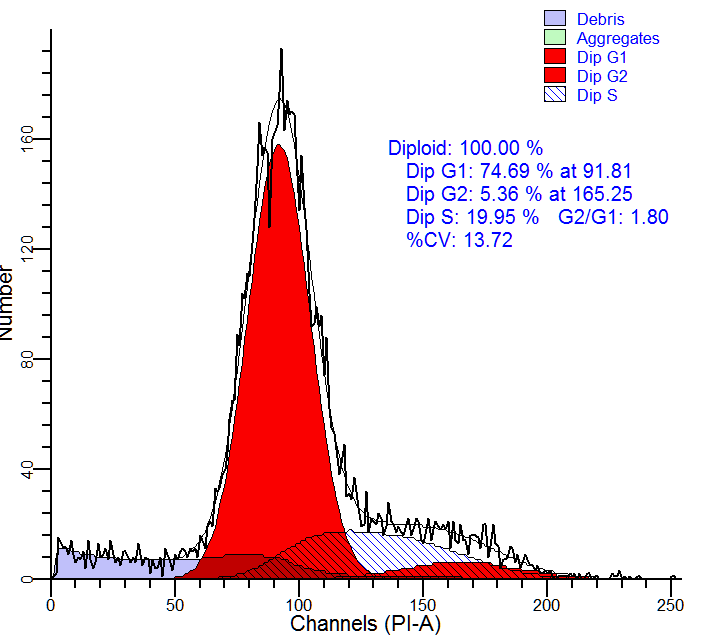


16 h

20 h


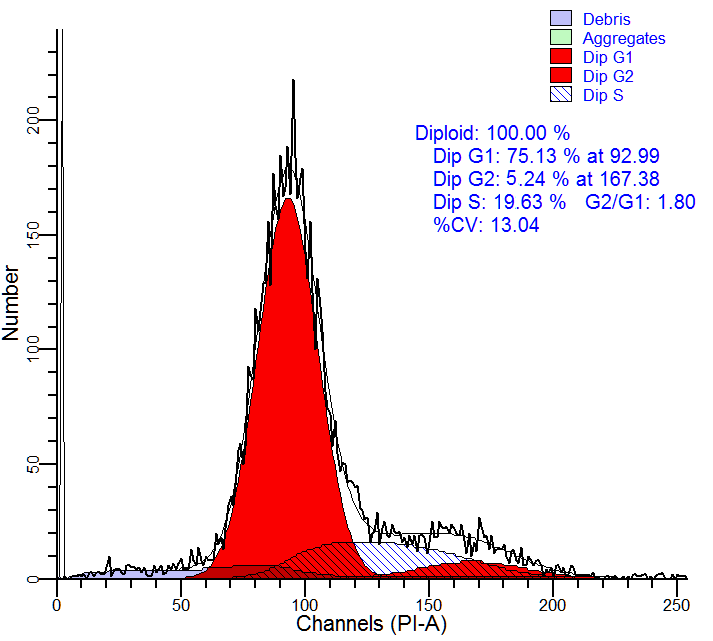


**Supplementary 3. Effect of starved serum on HeLa cells cycle.** HeLa **c**ells were starved without serum for 16 h, 18 h or 20 h, and then using PI staining assay.

In order to better understand the mechanism of growth inhibition exerted by Lm-PHB2 on HeLa cells, the percentage of G2/M phase cells in the presence of Lm-PHB2 protein was analyzed using PI staining. Compared to the PBS-treated group, the percentage of G2/M phase cells in rLm-PHB2-treated groups was increased, and in an rLm-PHB2 concentration-dependent manner.

PBS(0 μM rLm-PHB2)

0.625 μM rLm-PHB2

1.25 μM rLm-PHB2

2.5 μM rLm-PHB2


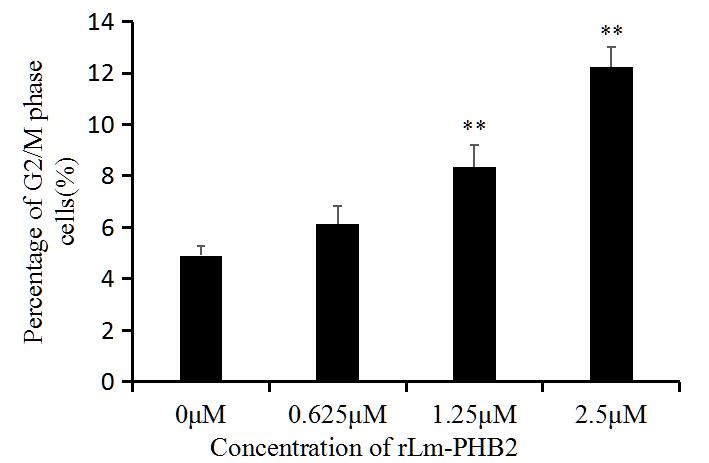


**Supplementary 4. Effect of Lm-PHB2 on the cell cycle.** HeLa cells were pretreated with PBS, 0.625 μM, 1.25 μM or 2.5 μM Lm-PHB2 at 37 ℃ for 48 h followed by staining with PI and flow cytometry analysis. The histogram compares the percentage of HeLa cells in the G2/M phase for Lm-PHB2-treated and PBS-treated groups. Data are the means ±SDs from three experiments. ‘**’ indicates significantly different from PBS-treated cells at the *P*<0.01 level.

Our results showed that compared to the PBS-treated group, the percentage of G2/M phase cells in Lm-PHB2-treated groups was increased, and in an Lm-PHB2 concentration-dependent manner.

1. ^*^ Qingwei Li to liqw@126.net [↑](#footnote-ref-2)
2. ^*^ Tiesong Li to sally_ts_li@163.com [↑](#footnote-ref-3)
